# Clinical Severity and Organ Dysfunction as Drivers of Mortality in Antimicrobial-Resistant *Acinetobacter baumannii* Pneumonia: A Retrospective Cohort Study

**DOI:** 10.3390/antibiotics15060578

**Published:** 2026-06-07

**Authors:** Ioana Adelina Stoian, Bianca Balas Maftei, Constantin Aleodor Costin, Radu Crișan-Dabija, Costin Damian, Robert Paval, Carmen Manciuc

**Affiliations:** 1 Grigore T. Popa University of Medicine and Pharmacy, 700115 Iasi, Romania; stirbu_ioana-adelina@d.umfiasi.ro (I.A.S.); radu.dabija@umfiasi.ro (R.C.-D.);; 2 Clinical Hospital of Pneumology, 700115 Iasi, Romania; 3 Research and Development Laboratory, 700115 Iasi, Romania; 4 ”Sfânta Parascheva” Clinical Hospital for Infectious Diseases, 700116 Iasi, Romania

**Keywords:** *Acinetobacter baumannii*, pneumonia, in-hospital mortality, sepsis, mechanical ventilation, acute kidney injury, inflammatory markers

## Abstract

**Background/Objectives:** *Acinetobacter baumannii* is a major cause of hospital-acquired and ventilator-associated pneumonia and is associated with high mortality among critically ill patients. Although antimicrobial resistance remains a major therapeutic challenge, the relative contribution of clinical severity, organ dysfunction, and laboratory parameters to patient outcomes requires further clarification. **Methods:** We conducted a retrospective single-center cohort study including 165 patients with microbiologically confirmed *A. baumannii* pneumonia admitted to the Clinical Hospital of Pneumology Iași, Romania, between 2019 and 2025. Clinical, laboratory, and outcome data were analyzed, and multivariable logistic regression was performed to identify independent predictors of in-hospital mortality. **Results:** In-hospital mortality was independently associated with older age (OR: 1.05 per year, 95% CI: 1.01–1.08, *p* = 0.005), sepsis (OR: 5.23, 95% CI: 1.93–16.5, *p* = 0.002), and mechanical ventilation (OR: 6.71, 95% CI: 3.02–15.6, *p* < 0.001). In exploratory analyses restricted to patients with available lactate measurements, lactate levels were not significantly associated with mortality, whereas acute kidney injury and dynamic renal deterioration were associated with increased mortality. Inflammatory markers, particularly neutrophil-to-lymphocyte ratio and C-reactive protein at 72 h, were significantly higher in non-survivors. **Conclusions:** These findings suggest that mortality in *A. baumannii* pneumonia is more closely associated with clinical severity, sepsis, respiratory failure, and evolving organ dysfunction than with isolated laboratory parameters. Early recognition of sepsis, acute kidney injury, and respiratory failure, together with serial assessment of inflammatory biomarkers, may support improved risk stratification in this high-risk population.

## 1. Introduction

*Acinetobacter baumannii (A. baumannii)* is a major cause of healthcare-associated infections, particularly hospital-acquired and ventilator-associated pneumonia, which most commonly affecting critically ill patients in intensive care units (ICUs) [1,2,3,4]. The clinical importance of this pathogen has increased significantly due to its remarkable ability to acquire resistance to multiple antimicrobial classes, leading to the global spread of multidrug-resistant (MDR) and carbapenem-resistant *A. baumannii* (CRAB). Accordingly, CRAB has been designated by the World Health Organization as a critical priority pathogen [5,6,7,8].

Infections caused by *A. baumannii* are associated with poor clinical outcomes, including prolonged hospitalization and high mortality rates, which have been reported to range between 30% and 60% in recent studies [9,10,11,12]. However, mortality in patients with *A. baumannii* pneumonia is influenced not only by antimicrobial resistance, but also by host-related factors, disease severity, and organ dysfunction [13]. Advanced age, mechanical ventilation, and sepsis have been consistently identified as major predictors of mortality in patients with severe pneumonia and critical illness [2,13,14]. The need for mechanical ventilation reflects respiratory failure and increased disease severity, whereas sepsis represents a dysregulated host response to infection and is strongly associated with adverse outcomes [2,14,15]. In addition, organ dysfunction, particularly acute kidney injury (AKI), has been associated with worse outcomes in critically ill patients, reflecting the systemic impact of severe infection [16,17,18].

Despite extensive research on antimicrobial resistance in *A. baumannii*, the relative contribution of clinical severity, organ dysfunction, inflammatory markers, and laboratory parameters to mortality in *A. baumannii* pneumonia remains incompletely understood. Inflammatory biomarkers are being increasingly used to assess disease severity and monitor clinical progression. Yet, their prognostic value may depend more on dynamic changes over time than on isolated baseline measurements [19,20]. Therefore, the aim of this study was to identify clinical and laboratory factors associated with mortality in patients with antimicrobial-resistant *A. baumannii* pneumonia, which may have the potential to support early risk stratification and inform clinical management.

### Statistical Analysis

All statistical analyses were performed using the programming language R (version 4.3). Continuous variables were assessed for normality using the Shapiro–Wilk test. Normally distributed variables are reported as mean ± standard deviation (SD), whereas non-normally distributed variables are summarized as median and interquartile range (IQR: Q1–Q3).

Group comparisons for continuous variables were performed using Student’s *t*-test when normality assumptions were met, and the Wilcoxon rank-sum test otherwise. Categorical variables were analyzed using Pearson’s chi-squared test or Fisher’s exact test, depending on expected cell counts. For univariate analysis, patients were stratified according to survival status. Analyses were performed using a complete-case approach, and missing values were excluded from the denominator for each individual variable.

To identify independent predictors of in-hospital mortality, logistic regression analysis was performed. Candidate predictors were initially evaluated in univariable models. The final multivariable model was intentionally kept parsimonious and included age, sepsis, mechanical ventilation, and initially active antibiotic therapy. Variables were selected based on clinical relevance and model parsimony.

Model performance was assessed using variance inflation factors (VIFs) to evaluate multicollinearity, the Hosmer–Lemeshow goodness-of-fit test was used for calibration, and the area under the receiver operating characteristic (ROC) curve (AUC) was used for discrimination. Results are reported as odds ratios (ORs) with 95% confidence intervals (CIs). A *p*-value < 0.05 was considered statistically significant. For analyses involving multiple comparisons, *p*-values were adjusted using the false discovery rate (FDR) method. Lactate-related analyses were considered exploratory because serum lactate measurements were only available in a subset of patients. Among patients with available lactate measurements, lactate was analyzed as a continuous variable and according to predefined thresholds of ≥2, ≥2.5, ≥2.8, and ≥3 mmol/L. Associations between lactate thresholds and in-hospital mortality were assessed using Fisher’s exact test, while lactate as a continuous predictor was evaluated using univariable logistic regression. ROC analysis was performed to assess the discriminative ability of lactate for in-hospital mortality. Additional exploratory analyses compared lactate levels and in-hospital mortality between septic and non-septic patients within the lactate-available subgroup.

Inflammatory biomarkers, including NLR and CRP at 24 and 72 h and their delta values, were additionally compared according to sepsis status using the available biomarker data. These comparisons were performed using the Wilcoxon rank-sum test.

## 2. Results

### 2.1. Baseline Characteristics of the Study Cohort

The median age of participants was 70 years (IQR: 62–77), and 64% of patients were male. The median BMI was 26.6 kg/m^2^ (IQR: 20.8–30.4). According to BMI category, 30% of patients were overweight, 29% had normal weight, 27% were obese, and 14% were underweight.

Most patients were from rural areas (57%), while 43% were from urban areas. Regarding the initial ward, most patients were admitted to pulmonology wards (43%) or ICUs (37%), followed by thoracic surgery (11%) and other wards (9.1%).

VAP was the most common pneumonia type, accounting for 43% of cases with available data, followed by HAP in 35% and CAP in 23%. Respiratory specimens were predominantly respiratory aspirates (82%), while sputum samples accounted for 18% of samples.

Most isolates were classified as MDR (87%), while 13% were XDR. Previous antibiotic exposure within the last 90 days was documented in 69% of patients with available data.

Comorbidities were common in the study population. Chronic obstructive pulmonary disease (COPD) was present in 47% of patients, hypertension in 46%, heart failure in 44%, chronic respiratory failure in 35%, malignancy in 22%, and diabetes mellitus in 17%. Sepsis was present in 31% of patients, while bacteremia was documented in 24%.

Mechanical ventilation was required in 71% of cases. Baseline laboratory parameters showed substantial variability. The median white blood cell count within the first 24 h was 12.79 × 10^9^/L (IQR: 9.33–17.23), while the median C-reactive protein (CRP) level was 93.9 mg/L (IQR: 25.5–172). The median neutrophil-to-lymphocyte ratio (NLR) was 11.49 (IQR: 5.42–21.48), and the median platelet count was 247.5 × 10^9^/L (IQR: 185–327.5). The mean hemoglobin value was 11.53 ± 2.35 g/dL. Renal function parameters showed a median creatinine level of 0.82 mg/dL (IQR: 0.59–1.21) and a median eGFR of 89 mL/min/1.73 m^2^ (IQR: 57–103).

Baseline characteristics of the study population are summarized in Table 1 and Table 2.

### 2.2. Association Between Lactate Levels and Mortality

**Figure 1:**
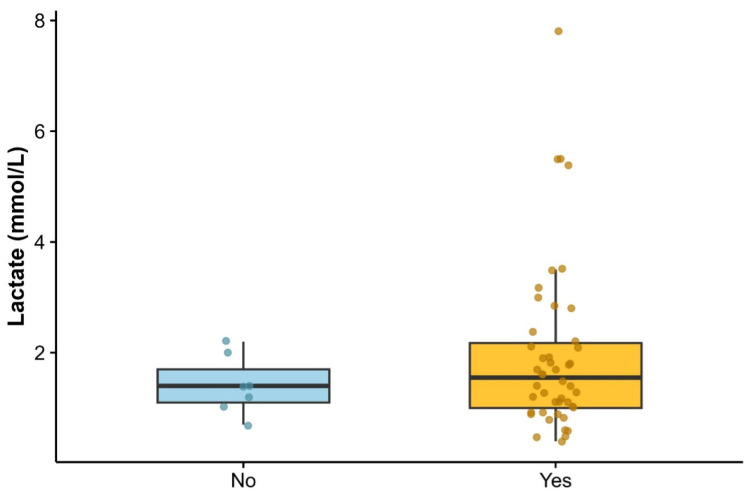
Lactate levels according to in-hospital mortality. Individual dots represent patient-level lactate values, boxplots show the median and interquartile range, and whiskers indicate the data range. Blue dots represent survivors, and orange dots represent non-survivors.

Among the 53 patients with available lactate measurements, lactate levels did not differ significantly according to in-hospital mortality status. Median lactate values were similar in patients who survived hospitalization and in those who died during hospitalization (1.40 [IQR: 1.00–2.00] vs. 1.55 [IQR: 1.00–2.20], *p* = 0.636). Similarly, when 30-day survival was used as the outcome, lactate levels were not significantly different between patients who survived and those who did not survive (1.60 [IQR: 1.00–2.20] vs. 1.30 [IQR: 1.00–1.40], *p* = 0.415). When lactate was analyzed using a threshold of 2 mmol/L, no significant association with in-hospital mortality was observed (86% vs. 88%, *p* > 0.999) (Table 3 and Figure 1).

Additional exploratory analyses using predefined lactate thresholds of 2.5, 2.8, and 3.0 mmol/L also failed to demonstrate significant associations with in-hospital mortality. For higher thresholds, all patients above the cut-off died during hospitalization; however, these results were affected by sparse data and quasi-complete separation and should therefore be interpreted cautiously. Likewise, lactate was not significantly associated with mortality when analyzed as a continuous variable (OR: 1.52, 95% CI: 0.77–4.92, *p* = 0.364). ROC analysis showed limited discriminative performance (AUC: 0.557), supporting the limited prognostic utility of lactate in this cohort. These findings should be interpreted as exploratory due to the limited number of patients with available lactate measurements.

### 2.3. Sepsis Subgroup Analyses

Among the 53 patients with available lactate measurements, median lactate levels were numerically higher in septic patients than in non-septic patients (1.65 vs. 1.40 mmol/L), although the difference did not reach statistical significance (*p* = 0.114). In-hospital mortality was high in both groups (95% vs. 81%, *p* = 0.218). These findings suggest that within this limited subgroup with available lactate measurements, sepsis was associated with a trend toward higher lactate levels and mortality, although statistical significance was not achieved, likely due to the small sample size.

### 2.4. Multivariable Analysis of In-Hospital Mortality

A multivariable logistic regression model was used to identify independent predictors of in-hospital mortality.

**Figure 2:**
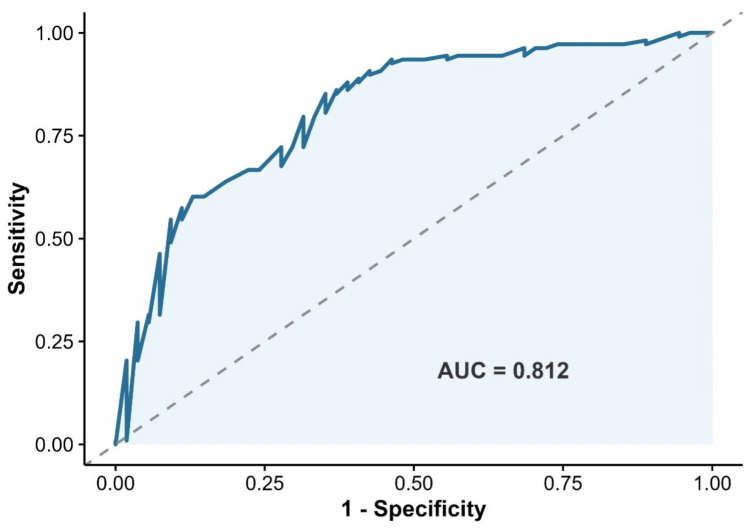
ROC curve of the multivariable mortality model. The solid blue line represents the ROC curve, the shaded area indicates the area under the curve (AUC), and the dashed grey line represents the no-discrimination reference line. The model showed good discriminatory performance (AUC = 0.812).

A total of 162 patients with complete data were included in the analysis, of whom 108 died during hospitalization and 54 survived. In the multivariable model, age was independently associated with in-hospital mortality (OR: 1.05, 95% CI: 1.01–1.08, *p* = 0.005). Sepsis was also significantly associated with increased mortality (OR: 5.23, 95% CI: 1.93–16.5, *p* = 0.002), as was the need for mechanical ventilation (OR: 6.71, 95% CI: 3.02–15.6, *p* < 0.001). Initial active antibiotic therapy was not significantly associated with in-hospital mortality after adjustment (OR: 1.38, 95% CI: 0.30–6.85, *p* = 0.7). Model performance was good, with an area under the receiver operating characteristic curve (AUC) of 0.812. Calibration was also satisfactory according to the Hosmer–Lemeshow test (*p* = 0.601). No significant multicollinearity was detected, as all variance inflation factors were close to 1 (Table 4 and Figure 2).

In unadjusted analyses, pneumonia type was significantly associated with in-hospital mortality in the univariable analysis. Mortality increased progressively across pneumonia categories, from 48.6% in patients with community-acquired pneumonia (CAP) to 62.5% in hospital-acquired pneumonia (HAP) and 81.2% in ventilator-associated pneumonia (VAP) (*p* = 0.002). Compared with CAP, VAP was associated with a significantly higher risk of death (OR: 4.55, 95% CI: 1.91–11.3, *p* < 0.001). However, after adjustment for age, sepsis, and mechanical ventilation in the multivariable model, pneumonia type was no longer significantly associated with in-hospital mortality. This suggests that the observed differences in mortality across pneumonia types were largely driven by underlying disease severity, particularly the need for mechanical ventilation.

To further explore potential determinants of mortality, renal parameters were evaluated in separate multivariable models. Creatinine at 72 h (OR: 1.84, 95% CI: 1.12–3.49, *p* = 0.035), eGFR at 72 h (OR: 0.98, 95% CI: 0.96–0.99, *p* < 0.001), and acute kidney injury (OR: 3.38, 95% CI: 1.36–9.13, *p* = 0.011) were each significantly associated with in-hospital mortality when evaluated independently. Given the strong collinearity between creatinine and eGFR, these variables were not included simultaneously in the same model.

### 2.5. Renal Function and Acute Kidney Injury and Mortality

**Figure 3:**
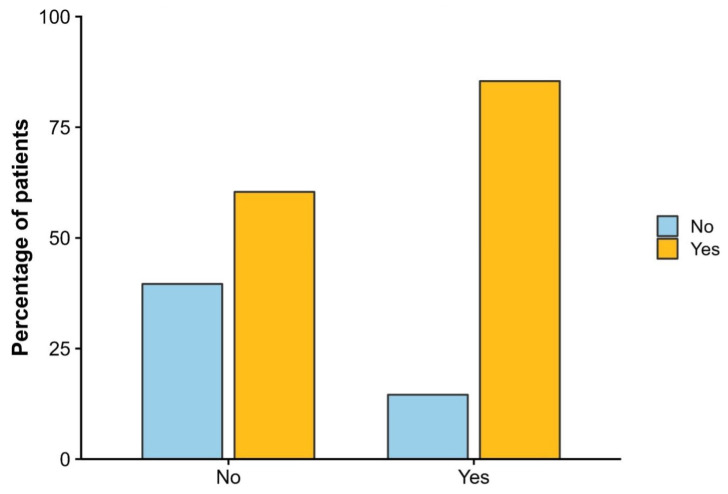
In-hospital mortality according to AKI status.

Renal dysfunction was common in the study population. Median creatinine was 0.82 mg/dL (IQR: 0.59–1.21) at 24 h and 0.87 mg/dL (IQR: 0.55–1.46) at 72 h, while median eGFR was 89 mL/min/1.73 m^2^ (IQR: 57–103) at 24 h and 85 mL/min/1.73 m^2^ (IQR: 46–103) at 72 h. An eGFR below 60 mL/min/1.73 m^2^ at any time point was observed in 44% of patients, and creatinine values above 1.5 mg/dL were documented in 35%. Acute kidney injury (AKI) was observed in 35% of patients. Patients with AKI had significantly higher in-hospital mortality compared to those without AKI (85% vs. 60%, *p* = 0.002), as well as lower 30-day survival (13% vs. 35%, *p* = 0.005). AKI was also associated with shorter hospital stays (12 [IQR 7–21] vs. 18 [IQR 11–26] days, *p* = 0.011), likely reflecting early mortality rather than faster clinical recovery. Additionally, patients with AKI had an increased need for ICU transfer (51% vs. 31%, *p* = 0.021) and a higher rate of mechanical ventilation (85% vs. 67%, *p* = 0.027) (Table 5 and Table 6 and Figure 3).

Additional analysis of dynamic renal parameters revealed significant differences between survivors and non-survivors. While creatinine levels at 24 h were not significantly different between groups, values at 72 h were significantly higher in non-survivors (1.19 vs. 0.61 mg/dL, *p* < 0.001). Similarly, eGFR at 72 h was significantly lower in non-survivors (67 vs. 100 mL/min/1.73 m^2^, *p* < 0.001), whereas no significant difference was observed at 24 h. Dynamic changes over time further highlighted these differences. Non-survivors showed an increase in creatinine (Δ +0.13 mg/dL), while survivors demonstrated a decrease (Δ −0.15 mg/dL, *p* < 0.001). Likewise, eGFR improved in survivors but declined in non-survivors (*p* < 0.001). Renal dysfunction criteria were also associated with mortality. Acute kidney injury was more common among non-survivors (44% vs. 17%, *p* = 0.002), and an eGFR below 60 mL/min/1.73 m^2^ at any time point was observed in 54% of non-survivors compared with 24% of survivors (*p* < 0.001) (Table 7 and Table 8).

### 2.6. Inflammatory Biomarkers and Outcome

**Figure 4:**
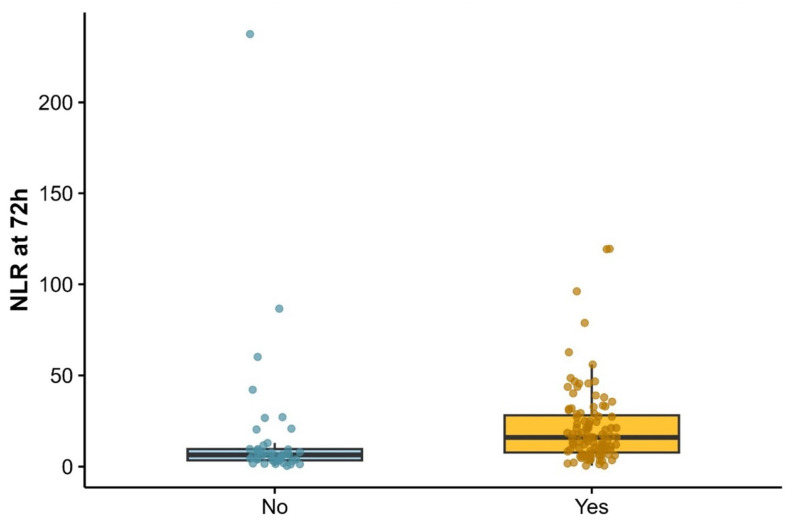
NLR at 72 h (sensitivity analysis, outliers removed). Individual dots represent patient-level NLR values, boxplots show the median and interquartile range, and whiskers indicate the data range after outlier removal. Blue dots represent survivors, and orange dots represent non-survivors.

**Figure 5:**
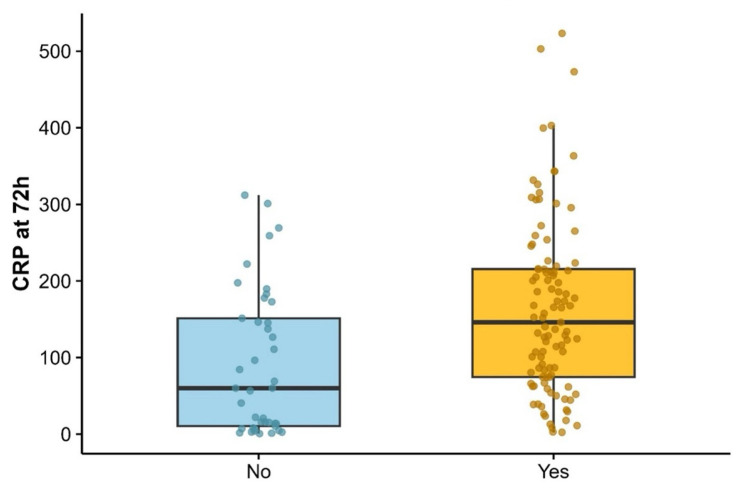
CRP at 72 h according to in-hospital mortality. Individual dots represent patient-level CRP values, boxplots show the median and interquartile range, and whiskers indicate the data range. Blue dots represent survivors, and orange dots represent non-survivors.

Inflammatory markers differed between survivors and non-survivors. Non-survivors had significantly higher neutrophil-to-lymphocyte ratios (NLRs) at both 24 h and 72 h. Median NLR at 24 h was 13 (IQR: 7–23) in non-survivors compared with 7 (IQR: 4–17) in survivors (*p* = 0.002). At 72 h, the difference was more pronounced, with a median NLR of 16 (IQR: 8–29) in non-survivors compared with 6 (IQR: 3–10) in survivors (*p* < 0.001). Similarly, C-reactive protein (CRP) and white blood cell count (WBC) at 72 h were significantly higher in non-survivors. In contrast, no significant differences were observed in CRP and WBC values at 24 h between the two groups (Table 9 and Figure 4 and Figure 5).

Additional analyses were performed to compare inflammatory biomarkers according to sepsis status in the cohort with available biomarker data. NLR at 24 h, NLR at 72 h, and ΔNLR did not differ significantly between septic and non-septic patients. In contrast, CRP levels were significantly higher among septic patients at both 24 and 72 h. CRP at 24 h was 132.60 mg/L in septic patients versus 65.40 mg/L in non-septic patients (*p* = 0.002), while CRP at 72 h was 165.10 mg/L versus 110.80 mg/L (*p* = 0.009). ΔCRP did not differ significantly between groups (Table 10).

### 2.7. Impact of Baseline Comorbidities on In-Hospital Mortality

The impact of baseline comorbidities on in-hospital mortality was further evaluated. Most comorbid conditions, including chronic obstructive pulmonary disease, diabetes mellitus, and cardiovascular disease, were similarly distributed between survivors and non-survivors and were not significantly associated with mortality. Malignancy was more common among non-survivors (27% vs. 11%, *p* = 0.036) and was associated with an increased risk of death in the univariable analysis (OR: 2.95, 95% CI: 1.21–8.34). However, this association was no longer significant after FDR correction for multiple testing, and no comorbidity remained statistically significant. Overall, these findings indicate that baseline comorbidities played a limited role in determining outcomes compared with acute markers of disease severity.

## 3. Discussion

In this retrospective cohort study of patients with *A. baumannii* pneumonia, we evaluated the association between clinical characteristics, antimicrobial resistance context, laboratory parameters, and treatment-related factors with in-hospital mortality. The main findings of our study indicate that mortality was primarily associated with markers of disease severity, including age, sepsis, and the need for mechanical ventilation. In contrast, lactate levels were not significantly associated with mortality. Additionally, acute kidney injury and persistent elevation of inflammatory markers, particularly NLR, CRP, and WBC at 72 h, were associated with worse outcomes, highlighting the multifactorial nature of prognosis in this high-risk population. Overall, our results suggest that outcomes in *A. baumannii* pneumonia are determined less by a single laboratory or microbiological parameter and more by the combined effect of host vulnerability, systemic infection severity, respiratory failure, and organ dysfunction. This is particularly relevant in cohorts dominated by multidrug-resistant organisms, where antimicrobial resistance represents an important therapeutic challenge, while clinical severity and organ dysfunction appear to be stronger determinants of patient outcomes [21,22].

### 3.1. Prognostic Value of Lactate

The role of lactate as a prognostic marker in severe infections has been extensively studied, particularly in the context of sepsis, where elevated levels are generally associated with increased mortality [15,23]. However, in exploratory analyses restricted to patients with available lactate measurements, lactate levels were not significantly associated with either in-hospital mortality or 30-day survival. Median lactate values were comparable between survivors and non-survivors, and a threshold-based analysis using 2 mmol/L failed to demonstrate a significant association with in-hospital death. Additional exploratory analyses using higher predefined thresholds (2.5, 2.8, and 3.0 mmol/L) also failed to identify significant associations with mortality. Although all patients above the higher lactate thresholds died, these analyses were limited by sparse data and zero survivors above the cut-off and therefore should be interpreted as exploratory rather than as evidence for clinically validated thresholds. Similarly, lactate was not significantly associated with mortality when analyzed as a continuous variable. ROC analysis showed limited discriminative ability (AUC: 0.557), indicating poor prognostic performance in this cohort. This finding may reflect the complex and heterogeneous nature of critically ill patients, in whom lactate levels can be influenced by multiple factors beyond tissue hypoperfusion, including comorbidities, organ dysfunction, and therapeutic interventions [24]. Moreover, a single lactate measurement may not adequately capture the dynamic metabolic response to infection, particularly in patients with prolonged hospitalization, ventilator-associated pneumonia, or advanced organ dysfunction [25].

Previous studies have reported conflicting results regarding the prognostic value of lactate in pneumonia and critically ill populations [26,27]. While some authors have demonstrated an association between elevated lactate levels and increased mortality, others have highlighted its limited specificity, particularly in heterogeneous ICU cohorts [24,27]. Our findings support the latter perspective, suggesting that lactate should be interpreted as part of a broader clinical assessment rather than as an isolated predictor of outcome, particularly in cohorts characterized by advanced disease severity and organ dysfunction.

### 3.2. Predictors of Mortality

Mortality in patients with *A. baumannii* pneumonia appears to be more closely associated with markers of disease severity than with microbiological factors alone [28,29,30]. In our study, older age, sepsis, and the need for mechanical ventilation were independently associated with in-hospital mortality, highlighting the central role of host-related and clinical factors in determining outcomes [31].

Advanced age has been consistently identified as a predictor of mortality in patients with severe infections and pneumonia, reflecting both decreased physiological reserve and the higher burden of comorbidities [1,13]. In our model, each additional year of age was associated with increased odds of in-hospital death, supporting the importance of age as a simple but clinically relevant risk stratification variable. Sepsis was also independently associated with mortality. This finding is expected, as sepsis represents a critical stage of infection characterized by a dysregulated host response and infection-related organ dysfunction [15]. In patients with *A. baumannii* pneumonia, the presence of sepsis likely reflects the transition from localized pulmonary infection to systemic disease, with subsequent hemodynamic instability, inflammatory amplification, and increased risk of multiorgan failure.

The need for mechanical ventilation showed the strongest association with mortality in the multivariable model. Mechanical ventilation is not only a marker of severe respiratory failure but also identifies a particularly vulnerable subgroup of patients with increased risk of ventilator-associated complications, prolonged ICU stay, secondary infections, and treatment failure [14,32]. Our findings are consistent with previous studies showing that respiratory failure requiring ventilatory support is one of the strongest predictors of poor outcomes in hospital-acquired and ventilator-associated pneumonia.

In the univariable analysis, pneumonia type was significantly associated with in-hospital mortality. In our cohort, mortality increased progressively from CAP to HAP and VAP, reaching 81.2% among patients with ventilator-associated pneumonia. This finding is consistent with previous literature showing that VAP caused by *A. baumannii* is associated with high mortality, particularly in critically ill patients requiring prolonged ventilatory support and ICU care [2,14]. However, in our analysis, pneumonia type was no longer independently associated with mortality after adjustment for age, sepsis, and mechanical ventilation. This suggests that the excess mortality observed in VAP was largely explained by the severity of illness and the need for organ support rather than by pneumonia classification alone. Similar observations have been reported in studies of severe hospital-acquired and ventilator-associated pneumonia, where mechanical ventilation, septic shock, and organ dysfunction were stronger determinants of outcome than infection category alone [1,26,31].

Similarly, when evaluating patient-related baseline characteristics, comorbidities were less strongly associated with mortality than acute markers of disease severity in our cohort. Although malignancy was more common among non-survivors and was associated with mortality in the univariable analysis, this association did not remain significant after correction for multiple testing. Previous studies have reported that comorbidities may contribute to poor outcomes in *A. baumannii* infections, particularly in older and critically ill patients, but their effect is often attenuated after accounting for acute severity markers such as sepsis, mechanical ventilation, and organ dysfunction [12,31]. In this context, our findings suggest that acute clinical deterioration had a stronger influence on mortality than baseline comorbidity burden. This is consistent with the broader critical care literature, where acute physiological derangement and organ failure are frequently more predictive of short-term mortality than chronic comorbid conditions alone [21,22]. Our findings partially differ from previous evidence. A meta-analysis by Du et al. reported higher baseline severity scores, including Pitt bacteremia score, among non-survivors with CRAB infection. However, that study included heterogeneous observational cohorts, predominantly bacteremia populations, whereas our analysis specifically focused on antimicrobial-resistant *A. baumannii* pneumonia. In our cohort, dynamic organ dysfunction during hospitalization appeared to have greater prognostic relevance than isolated baseline severity indicators [33].

Interestingly, initially active antibiotic therapy was not independently associated with mortality after adjustment. This finding should not be interpreted as evidence that antimicrobial therapy is not important. While early and appropriate antimicrobial therapy is generally considered a cornerstone of sepsis management, its effect may reflect confounding by disease severity or limitations of retrospective data [15,23]. This finding further supports the concept that, in critically ill patients with *A. baumannii* pneumonia, the prognostic impact of clinical severity may be greater than that of individual treatment-related variables, particularly when treatment effects are influenced by timing, disease stage, and baseline mortality risk. Taken together, these findings indicate that risk stratification in *A. baumannii* pneumonia should integrate demographic vulnerability, sepsis, respiratory failure, and organ dysfunction, given that in our cohort, age, sepsis, and mechanical ventilation showed stronger independent associations with mortality than resistance phenotype or antibiotic selection.

### 3.3. Renal Dysfunction and Acute Kidney Injury

Renal dysfunction and acute kidney injury are well-recognized markers of poor prognosis in critically ill patients. In our study, AKI was common and was significantly associated with increased in-hospital mortality, lower 30-day survival, higher need for ICU transfer, and more frequent mechanical ventilation. These findings suggest that AKI was more commonly observed in patients with more severe systemic illness.

The association between AKI and mortality is biologically plausible. AKI may reflect hemodynamic instability, systemic inflammation, endothelial dysfunction, nephrotoxic exposure, and multiorgan involvement during severe infection [16,34]. Previous studies have demonstrated that AKI is independently associated with increased mortality in patients with sepsis and severe bacterial infections, with renal impairment frequently representing a component of progressive multiorgan dysfunction [16,34]. In addition, renal dysfunction can complicate antimicrobial management by limiting therapeutic options, requiring dose adjustment, and increasing the risk of drug toxicity. This is particularly relevant in infections caused by MDR *A. baumannii*, where treatment regimens may include potentially nephrotoxic agents such as colistin or high-dose ampicillin-sulbactam.

In our cohort, patients with AKI had a shorter hospital stay compared with those without AKI. This finding should not be interpreted as a favorable outcome. In the context of significantly higher mortality, shorter hospitalization likely reflects earlier in-hospital death among patients with more severe disease rather than faster recovery. Although AKI was not included as an independent predictor in the final multivariable model to preserve a parsimonious model and avoid overfitting, its strong association with adverse outcomes remains clinically meaningful. These findings support the importance of early renal monitoring, prevention of nephrotoxic exposure where possible, optimization of hemodynamic status, and careful adjustment of antimicrobial therapy in patients with severe *A. baumannii* pneumonia [13].

The emergence of significant differences at 72 h, together with divergent trends in creatinine and eGFR between survivors and non-survivors, supports the concept that renal deterioration may reflect ongoing disease progression rather than pre-existing dysfunction alone. In the additional multivariable models, creatinine at 72 h, eGFR at 72 h, and AKI remained independently associated with in-hospital mortality, further supporting renal dysfunction as a clinically relevant prognostic domain. This observation is consistent with the broader understanding of acute kidney injury as a component of evolving multiorgan failure in severe infections. Previous studies have similarly shown that renal dysfunction and AKI are strongly associated with mortality in critically ill patients, particularly in the context of sepsis and severe infections [28,35]. Comparable findings have been reported in patients with multidrug-resistant Gram-negative infections, where organ dysfunction, including renal impairment, represents a key determinant of outcome [31]. Moreover, dynamic changes in renal function may better reflect the progression of organ failure and overall disease severity than baseline values alone.

### 3.4. Inflammatory Biomarkers and Disease Progression

Inflammatory biomarkers are widely used to assess disease severity and monitor progression in patients with severe infections. In our study, inflammatory markers differed between survivors and non-survivors, particularly when assessed dynamically. NLR was significantly higher in non-survivors at both 24 and 72 h, while CRP and WBC showed clearer differences at 72 h.

These findings suggest that single early inflammatory marker measurements may not have uniform prognostic value, whereas their persistence or increase over time, particularly at 72 h, may better reflect the clinical trajectory of the patient. Similar observations have been reported in previous studies, where dynamic changes in inflammatory markers were shown to be more informative than single measurements at admission [19,36,37].

NLR, in particular, has emerged as a simple and accessible marker associated with severity and mortality in various infectious diseases, including pneumonia [36,38,39]. Likewise, persistently elevated CRP levels have been associated with ongoing inflammation and worse outcomes in critically ill patients [19].

Additional exploratory analyses according to sepsis status showed significantly higher CRP levels at both 24 and 72 h among septic patients, whereas NLR values did not differ significantly between septic and non-septic groups. These findings are consistent with the established role of CRP as a marker of systemic inflammation and disease severity in sepsis [19], as defined by the Sepsis-3 framework [25]. The absence of significant differences in NLR suggests that inflammatory biomarkers may have heterogeneous performance depending on the clinical context and patient population.

Taken together, our results support the value of serial biomarker assessment as a tool for monitoring disease progression, rather than relying solely on initial values. Although antimicrobial resistance is a defining characteristic of *A. baumannii*, our findings suggest that these markers reflect underlying disease progression rather than acting as standalone determinants of outcome [40,41]. This highlights the need for integrated approaches that combine antimicrobial stewardship with early recognition of disease severity and organ dysfunction in the management of resistant infections [40,41]. These findings should be interpreted as hypothesis-generating and require validation in larger prospective studies.

### 3.5. Clinical Implications

Our findings have several clinical implications. Early assessment of disease severity, including sepsis, the need for mechanical ventilation, and renal dysfunction may support risk stratification in patients with *A. baumannii* pneumonia, as these factors were more strongly associated with mortality than isolated laboratory parameters.

In this context, lactate values should be interpreted with caution. In our cohort, lactate showed limited prognostic utility and should be interpreted within the broader clinical context.

At the same time, the dynamic evolution of inflammatory biomarkers may provide clinically relevant information regarding disease progression. Persistent elevation of NLR, CRP, and WBC at 72 h may help identify patients requiring closer monitoring and timely reassessment, particularly when interpreted alongside clinical severity and organ dysfunction.

Although antimicrobial resistance remains a defining feature of *A. baumannii*, our findings suggest that optimal management should not focus exclusively on microbiological resistance patterns. Instead, an integrated approach combining timely antimicrobial therapy, early sepsis management, respiratory support, renal monitoring and organ support is required, recognizing that clinical context and disease progression play a central role in determining patient outcomes.

## 4. Study Limitations

This study has several limitations that should be acknowledged. Its retrospective single-center design may introduce selection bias and limit the generalizability of the findings. However, it provides real-world data from a high-burden setting in Eastern Europe, which remains underrepresented in the literature. Some analyses were limited by missing data, particularly regarding lactate measurements, resulting in a reduced sample size for lactate-related analyses. Therefore, the lactate-related findings should be interpreted as exploratory.

Although multiple clinical, microbiological, and laboratory variables were evaluated, the multivariable model was intentionally restricted to a limited number of predictors to avoid overfitting. As a result, not all potentially relevant factors, including renal dysfunction, inflammatory biomarkers, and detailed treatment-related variables, could be incorporated simultaneously into a single comprehensive model. Therefore, the results of the multivariable analyses should be interpreted as targeted models evaluating selected prognostic domains rather than as a complete explanatory model of mortality.

In addition, the study was not designed to evaluate the comparative effectiveness of specific antimicrobial regimens, and inflammatory biomarkers were only assessed at a limited number of time points. Larger multicenter prospective studies are needed to validate these findings and to better define the interaction between clinical severity, organ dysfunction, antimicrobial therapy, and outcomes in patients with *A. baumannii* pneumonia.

## 5. Materials and Methods

The study included 165 patients with microbiologically confirmed *A. baumannii* pneumonia diagnosed at the Clinical Hospital of Pneumology Iași, Romania, between 2019 and 2025. Given the retrospective observational design of the study, clinical, microbiological, laboratory, and outcome data were extracted from medical records. The study was approved by the institutional review board of the Clinical Hospital of Pneumology Iași (approval No. 125/26 June 2025) and the Ethics Committee of Grigore T. Popa University of Medicine and Pharmacy Iași (approval No. 707/31 January 2026). The research adhered to the principles outlined in the Declaration of Helsinki for research involving human subjects.

**Figure 6:**
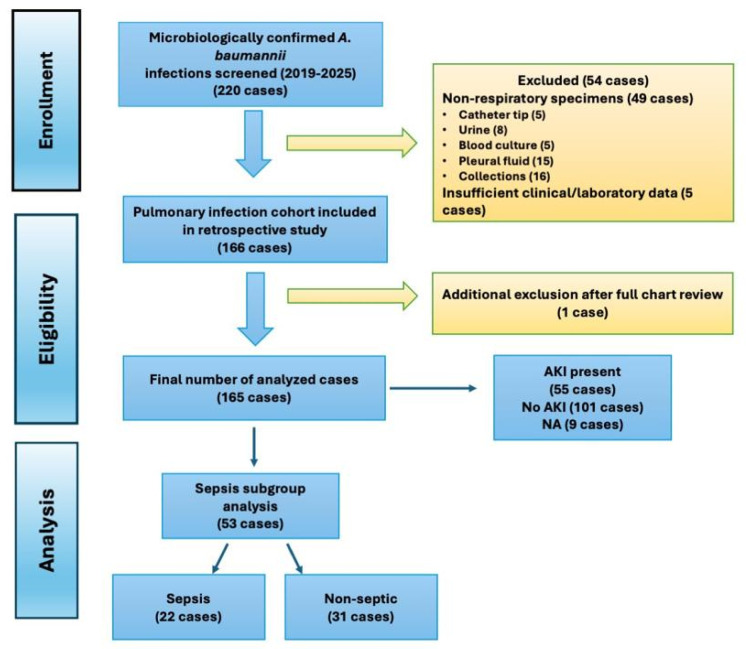
STROBE flow diagram of patient selection and analytic subgroups. NA—data not available.

A total of 220 patients with microbiologically confirmed *A. baumannii* infection were initially screened. Patients were excluded if *A. baumannii* was isolated exclusively from non-respiratory sources, if the criteria for pneumonia were not fulfilled, or if clinical or laboratory data were incomplete for the variables of interest. After applying the exclusion criteria, 165 patients with confirmed pulmonary infection were included in the final analysis. Patients were eligible if they had microbiological confirmation of *A. baumannii* from respiratory specimens, together with clinical and radiological findings consistent with pneumonia (Figure 6). Pneumonia was defined as the presence of a new or progressive pulmonary infiltrate on chest imaging, together with compatible clinical features such as fever or hypothermia, leukocytosis or leukopenia, purulent respiratory secretions, worsening respiratory status, or increased oxygen requirements. Microbiological confirmation was based on respiratory specimens, mainly respiratory aspirates and sputum, obtained according to clinical indication and patient status. The index date was defined as the date of the first positive respiratory culture for *A. baumannii* associated with clinical and radiological criteria of pneumonia. When multiple positive respiratory cultures were available during the same hospitalization, only the first episode fulfilling the pneumonia criteria was considered for analysis (Figure 1).

Pneumonia was classified as community-acquired pneumonia (CAP), hospital-acquired pneumonia (HAP), or ventilator-associated pneumonia (VAP) according to established ATS/IDSA guideline definitions [42,43]. CAP was defined as pneumonia present at admission or diagnosed within the first 48 h of hospitalization. HAP was defined as pneumonia occurring 48 h or more after hospital admission in patients not receiving invasive mechanical ventilation at pneumonia onset. VAP was defined as pneumonia developing more than 48 h after endotracheal intubation and initiation of mechanical ventilation.

Clinical, microbiological, laboratory, and outcome data were retrospectively extracted from medical records. Collected variables included demographic characteristics, comorbidities, ICU admission, requirement for mechanical ventilation, presence of sepsis, microbiological data including antimicrobial susceptibility and resistance profile, and antibiotic therapy (including adequacy and timing). Routine laboratory parameters, including white blood cell count (WBC), C-reactive protein (CRP), neutrophil-to-lymphocyte ratio (NLR), hemoglobin, platelet count, creatinine, estimated glomerular filtration rate (eGFR), and serum lactate, when available, were recorded at 24 and 72 h as part of routine clinical assessment. Serum lactate measurements were obtained from venous or arterial blood samples according to clinical indication and analyzed using the standard automated biochemical methods available in the hospital laboratory. Body mass index (BMI) was calculated as weight in kilograms divided by height in meters squared (kg/m^2^). Patients were classified according to BMI categories as underweight (BMI < 18.5 kg/m^2^), normal weight (BMI 18.5–24.9 kg/m^2^), overweight (BMI 25.0–29.9 kg/m^2^), or obese (BMI ≥ 30.0 kg/m^2^) [44]. Clinical outcomes included in-hospital mortality and 30-day survival. Thirty-day survival was assessed from the index date. Analyses were performed using a complete-case approach, and patients with missing data for specific variables were excluded from the respective analyses. Lactate-related analyses were considered exploratory because serum lactate measurements were only available in a subset of patients. Among patients with available lactate values, lactate was analyzed as a continuous variable and according to predefined thresholds of ≥2, ≥2.5, ≥2.8, and ≥3 mmol/L. Associations with in-hospital mortality were assessed using univariable logistic regression and Fisher’s exact test, as appropriate. Receiver operating characteristic (ROC) analysis was performed to evaluate the discriminative ability of lactate for in-hospital mortality. Additional exploratory analyses compared lactate levels and in-hospital mortality between septic and non-septic patients within the lactate-available subgroup. Inflammatory biomarkers, including NLR and CRP at 24 and 72 h and their delta values, were additionally compared according to sepsis status in the full cohort using available biomarker data.

Identification of *A. baumannii* isolates was performed according to the routine protocol of the hospital microbiology laboratory using matrix-assisted laser desorption/ionization time-of-flight mass spectrometry (MALDI-TOF MS; Bruker Daltonics, Bremen, Germany). Antimicrobial susceptibility testing was performed using the VITEK^®^ 2 automated system (bioMérieux, Marcy-l′Étoile, France). Susceptibility results were interpreted according to the European Committee on Antimicrobial Susceptibility Testing (EUCAST) clinical breakpoints and recommendations that were valid at the time of testing [45]. Multidrug-resistant (MDR) and extensively drug-resistant (XDR) phenotypes were defined according to standard international criteria based on non-susceptibility across antimicrobial categories [46].

Sepsis was identified based on clinical documentation and available laboratory and organ dysfunction parameters consistent with Sepsis-3 criteria [25]. Treatment was considered initially active when at least one administered antimicrobial agent showed in vitro activity against the isolated *A. baumannii* strain according to susceptibility testing results [40].

Renal dysfunction was defined as an estimated glomerular filtration rate (eGFR) <60 mL/min/1.73 m^2^. eGFR values were extracted from laboratory reports and were interpreted according to the routine method used by the hospital laboratory. Acute kidney injury (AKI) was defined according to KDIGO criteria [47]. For dynamic renal assessment, creatinine and eGFR values were evaluated at 24 h and 72 h when available. Delta values were calculated as the difference between 72-h and 24-h measurements. The primary outcome was in-hospital mortality, while 30-day survival was assessed as a secondary outcome.

## 6. Conclusions

In patients with *A. baumannii* pneumonia, in-hospital mortality was independently associated with older age, sepsis, and the need for mechanical ventilation, underscoring the important role of clinical severity and organ dysfunction in determining outcomes. In exploratory analyses restricted to patients with available lactate measurements, lactate levels were not significantly associated with mortality, suggesting that lactate should be interpreted within the broader clinical context rather than as an isolated prognostic marker.

AKI and impaired renal function at 72 h emerged as important prognostic factors and were associated with worse outcomes, supporting the importance of dynamic disease progression in this population. Persistent inflammatory activity was also observed among patients with adverse outcomes, although its independent prognostic value appeared less consistent. Mortality increased progressively from CAP to HAP and VAP; however, pneumonia type was no longer independently associated with outcome after adjustment for severity markers, suggesting that excess mortality was largely explained by respiratory failure and systemic illness rather than pneumonia classification alone.

Overall prognosis appears to be largely associated with the severity of illness, respiratory failure, renal dysfunction, and the clinical course rather than with microbiological characteristics alone. These findings suggest that mortality in *A. baumannii* pneumonia is more closely associated with clinical severity and evolving organ dysfunction than with isolated laboratory parameters, although persistently elevated inflammatory markers such as CRP, NLR, and WBC at 72 h were also associated with poor outcomes.

## Figures and Tables

**Table 1:** Baseline characteristics of the study population.

Domain	Characteristic		*N* (%)	NA
Demographics	Age, years		70 (62–77)	0
	Female		60 (36%)	0
	Male		105 (64%)	0
	BMI		26.60 (20.80–30.40)	0
	BMI category	Normal	48 (29%)	0
		Overweight	50 (30%)	
		Obesity	44 (27%)	
		Underweight	23 (14%)	
	Residence type	Rural	94 (57%)	0
		Urban	71 (43%)	
Clinical setting	Initial ward	ICU	61 (37%)	0
		Pulmonology	71 (43%)	
		Thoracic surgery	18 (11%)	
		Another ward	15 (9.1%)	
	Pneumonia type	CAP	37 (23%)	3
		HAP	56 (35%)	
		VAP	69 (43%)	
Microbiology	Specimen type	Aspirate	135 (82%)	1
		Sputum	29 (18%)	
	MDR/XDR profile	MDR	141 (87%)	2
		XDR	22 (13%)	
Comorbidities	COPD		73 (47%)	9
	Chronic respiratory failure		54 (35%)	9
	Diabetes		27 (17%)	9
	Heart failure		69 (44%)	9
	Hypertension		72 (46%)	8
	Malignancy		35 (22%)	6

Data are presented as median (IQR) for continuous variables and as *N* (%) for categorical variables. Percentages were calculated among patients with available data. NA—number of patients with missing data; BMI—body mass index; ICU—intensive care unit; CAP—community-acquired pneumonia; HAP—hospital-acquired pneumonia; VAP—ventilator-associated pneumonia; MDR—multidrug-resistant; XDR—extensively drug-resistant; COPD—chronic obstructive pulmonary disease.

**Table 2:** Disease severity, treatment history and laboratory parameters.

Domain	Characteristic	*N* (%)	NA
Severity markers	Sepsis	50 (31%)	2
	Bacteremia	37 (24%)	8
	Mechanical ventilation	117 (71%)	1
	Ventilation days	6 (0–15)	3
	ICU transfer	58 (36%)	3
Treatment history	Antibiotics in the last 90 days	96 (69%)	25
Laboratory parameters	WBC at 24 h, ×10^9^/L	12.79 (9.33–17.23)	5
	Creatinine at 24 h, mg/dL	0.82 (0.59–1.21)	2
	NLR at 24 h	11.49 (5.42–21.48)	5
	Hemoglobin at 24 h, g/dL	11.53 ± 2.35	5
	CRP at 24 h, mg/L	93.90 (25.50–172.00)	6
	eGFR at 24 h, mL/min/1.73 m^2^	89 (57–103)	2
	Platelets at 24 h, ×10^9^/L	247.50 (185.00–327.50)	5

Data are presented as median (IQR) for non-normally distributed continuous variables, mean ± SD for normally distributed continuous variables, and *N* (%) for categorical variables. Percentages were calculated among patients with available data. NA—number of patients with missing data; ICU—intensive care unit; WBC—white blood cell count; NLR—neutrophil-to-lymphocyte ratio; CRP—C-reactive protein; eGFR—estimated glomerular filtration rate.

**Table 3:** Lactate levels and mortality outcomes.

Characteristic	Comparison Group 1	Comparison Group 2	*p*-Value
In-hospital mortality	Survivors (*N* = 7)	Non-survivors (*N* = 46)	
Lactate, mmol/L	1.40 (1–2)	1.55 (1–2.20)	0.636
30-day survival	No (*N* = 47)	Yes (*N* = 6)	
Lactate, mmol/L	1.60 (1–2.20)	1.30 (1–1.40)	0.415
Lactate threshold analysis	<2 mmol/L (*N* = 37)	≥2 mmol/L (*N* = 16)	
In-hospital death, *N* (%)	32 (86%)	14 (88%)	>0.999

Data are presented as median (IQR) for lactate values and as *N* (%) for categorical variables. Lactate levels were compared using the Wilcoxon rank-sum test. The comparison of in-hospital mortality according to lactate category was performed using Fisher’s exact test.

**Table 4:** Multivariable logistic regression model for in-hospital mortality.

Variable	OR	95% CI	*p*-Value
Age (per year)	1.05	1.01–1.08	0.005
Sepsis (Yes vs. No)	5.23	1.93–16.5	0.002
Mechanical ventilation (Yes vs. No)	6.71	3.02–15.6	<0.001
Initially active antibiotic therapy (Yes vs. No)	1.38	0.30–6.85	0.700

Data are presented as odds ratios (ORs) with 95% confidence intervals (CIs). The model included 162 complete cases (108 deaths, 54 survivors). Model discrimination was good (AUC = 0.812). Calibration was satisfactory according to the Hosmer–Lemeshow test (*p* = 0.601). No significant multicollinearity was detected (variance inflation factors were close to 1).

**Table 5:** Renal function parameters in the whole cohort.

Characteristic	Whole Cohort *N* = 165	NA
Creatinine at 24 h, mg/dL	0.82 (0.59–1.21)	2
Creatinine at 72 h, mg/dL	0.87 (0.55–1.46)	8
eGFR at 24 h, mL/min/1.73 m^2^	89 (57–103)	2
eGFR at 72 h, mL/min/1.73 m^2^	85 (46–103)	8
Acute kidney injury	55 (35%)	9
eGFR <60 mL/min/1.73 m^2^ at any time	73 (44%)	0
Creatinine >1.5 mg/dL at any time	58 (35%)	0
eGFR decrease ≥20%	45 (29%)	9
BUN/creatinine ratio at 24 h	29 (21–37)	2
BUN/creatinine ratio at 72 h	32 (23–41)	8

Data are reported as median (IQR) for continuous variables and *N* (%) for categorical variables. NA—number of patients with missing data; eGFR—estimated glomerular filtration rate; BUN—blood urea nitrogen.

**Table 6:** Renal function parameters according to in-hospital mortality.

Characteristic	Variable	Survivors (*N* = 54)	Non-Survivors (*N* = 111)	Missing (S/NS)	*p*-Value
Serial measurements	Creatinine at 24 h, mg/dL	0.79 (0.56–1.07)	0.90 (0.61–1.32)	2/0	0.172
	Creatinine at 72 h, mg/dL	0.61 (0.46–0.93)	1.19 (0.58–1.85)	5/3	<0.001
	Delta creatinine, mg/dL	−0.15 (−0.39–0.18)	0.13 (−0.24–0.86)	6/3	<0.001
	eGFR at 24 h, mL/min/1.73 m^2^	96 (65–108)	87 (54–100)	2/0	0.070
	eGFR at 72 h, mL/min/1.73 m^2^	100 (80–115)	67 (33–98)	5/3	<0.001
	Delta eGFR, mL/min/1.73 m^2^	6 (−8–24)	−6 (−40–14)	6/3	<0.001
	BUN/creatinine ratio at 24 h	28 (19–36)	29 (22–37)	2/0	0.281
	BUN/creatinine ratio at 72 h	30 (20–40)	33 (24–42)	5/3	0.174
Renal dysfunction criteria	AKI, *n* (%)	8 (17%)	47 (44%)	6/3	0.002
	eGFR <60 mL/min/1.73 m^2^ at any time, *n* (%)	13 (24%)	60 (54%)	0/0	<0.001
	Creatinine >1.5 mg/dL at any time, *n* (%)	12 (22%)	46 (41%)	0/0	0.024
	eGFR drop ≥20% from baseline, *n* (%)	7 (15%)	38 (35%)	6/3	0.015

Data are reported as median (IQR) for continuous variables and *n* (%) for categorical variables. Missing = number of missing values (survivors/non-survivors; S/NS). Delta values = difference between 72 h and 24 h measurements. AKI = acute kidney injury; eGFR = estimated glomerular filtration rate; BUN = blood urea nitrogen; IQR = interquartile range. Percentages and summary statistics were calculated after excluding missing values for each variable.

**Table 7:** Multivariable logistic regression models with renal predictors.

Characteristic	Base Model OR (95% CI)	*p*-Value	+ Creatinine at 72 h OR (95% CI)	*p*-Value	+ eGFR at 72 h OR (95% CI)	*p*-Value	+ AKI OR (95% CI)	*p*-Value
Age, per year increase	1.05 (1.01–1.08)	0.005	1.04 (1.00–1.07)	0.034	1.01 (0.98–1.05)	0.500	1.04 (1.01–1.08)	0.011
Sepsis	5.23 (1.93–16.5)	0.002	5.57 (2.02–17.8)	0.002	6.97 (2.42–23.5)	<0.001	5.77 (2.06–18.8)	0.002
Mechanical ventilation	6.71 (3.02–15.6)	<0.001	4.46 (1.92–10.7)	<0.001	4.38 (1.84–10.8)	<0.001	4.61 (1.98–11.2)	<0.001
Initially active antibiotic therapy	1.38 (0.30–6.85)	0.700	1.27 (0.27–6.26)	0.800	1.25 (0.26–6.56)	0.800	1.37 (0.29–6.84)	0.700
Creatinine at 72 h, mg/dL	—	—	1.84 (1.12–3.49)	0.035	—	—	—	—
eGFR at 72 h, mL/min/1.73 m^2^	—	—	—	—	0.98 (0.96–0.99)	<0.001	—	—
AKI	—	—	—	—	—	—	3.38 (1.36–9.13)	0.011

OR = odds ratio; CI = confidence interval; AKI = acute kidney injury; eGFR = estimated glomerular filtration rate. Reference categories: Sepsis—No; Mechanical ventilation—No; Initially active antibiotic therapy—No; AKI—No—indicates that variable was not included in that model.

**Table 8:** Clinical outcomes according to acute kidney injury.

Characteristic	No AKI *N* = 101	Yes, AKI *N* = 55	NA No AKI/NA Yes AKI	*p*-Value
In-hospital death	61 (60%)	47 (85%)	0/0	0.002
30-day survival	35 (35%)	7 (13%)	1/0	0.005
Hospital stay, days	18 (11–26)	12 (7–21)	1/0	0.011
ICU transfer	30 (31%)	28 (51%)	3/0	0.021
Mechanical ventilation	68 (67%)	46 (85%)	0/1	0.027

Data are presented as median (IQR) for continuous variables and as *N* (%) for categorical variables, as appropriate. Percentages were calculated among patients with available data. Group comparisons were performed using the Wilcoxon rank-sum test or Pearson’s chi-square test, as appropriate. NA—number of patients with missing data; AKI—acute kidney injury; ICU—intensive care unit.

**Table 9:** Inflammatory biomarkers according to in-hospital mortality.

Characteristic	Survivors *N* = 54	Non-Survivors *N* = 111	NA Survivors/ NA Non-Survivors	*p*-Value
NLR at 24 h	7.00 (4.00–17.00)	13.00 (7.00–23.00)	3/2	0.002
NLR at 72 h	6.00 (3.00–10.00)	16.00 (8.00–29.00)	10/7	<0.001
ΔNLR	−1.00 (−9.00–2.00)	2.00 (−5.00–13.00)	11/9	0.100
CRP at 24 h, mg/L	67.00 (17.00–144.00)	100.00 (36.00–179.00)	4/2	0.135
CRP at 72 h, mg/L	60.00 (10.00–151.00)	146.00 (74.00–216.00)	13/8	<0.001
WBC at 24 h, ×10^9^/L	11.00 (9.00–15.00)	13.00 (10.00–19.00)	3/2	0.050
WBC at 72 h, ×10^9^/L	12.00 (10.00–18.00)	16.00 (11.00–25.00)	10/7	0.011

Data are presented as median (IQR). Group comparisons were performed using the Wilcoxon rank-sum test. NA—number of patients with missing data; ΔNLR represents the change in NLR between 24 and 72 h. NLR—neutrophil-to-lymphocyte ratio; CRP—C-reactive protein; WBC—white blood cell count.

**Table 10:** Inflammatory biomarkers according to sepsis status in the cohort with available biomarker data.

Characteristic	Non-Septic *N* = 113	Septic *N* = 50	Missing Non-Septic/Septic	*p*-Value
NLR at 24 h	11.47 (5.36–21.95)	11.54 (5.46–20.55)	5/0	0.853
NLR at 72 h	10.85 (5.72–23.21)	13.05 (5.91–29.00)	14/3	0.299
ΔNLR	1.19 (−8.14–6.99)	1.19 (−3.27–13.59)	17/3	0.251
CRP at 24 h, mg/L	65.40 (16.90–150.50)	132.60 (64.50–229.80)	6/0	0.002
CRP at 72 h, mg/L	110.80 (31.50–189.60)	165.10 (74.60–259.00)	18/3	0.009
ΔCRP	4.40 (−49.40–120.20)	26.50 (−80.80–75.50)	21/3	0.899

Data are presented as median (IQR). Group comparisons were performed using the Wilcoxon rank-sum test. Missing values were excluded from the denominator for each individual variable. NLR = neutrophil-to-lymphocyte ratio; CRP = C-reactive protein; Δ = change between 24 h and 72 h.

## Data Availability

The anonymized dataset supporting the findings of this study is available from the corresponding author upon reasonable request and subject to institutional approval. The data are not publicly available due to privacy and ethical restrictions related to patient-level clinical data. All personally identifiable information has been removed to preserve patient confidentiality.

## Abbreviations

*A. baumannii*	Acinetobacter baumannii
AKI	Acute Kidney Injury
AUC	Area Under the Curve
BMI	Body Mass Index
BUN/Cr ratio	Blood Urea Nitrogen to Creatinine ratio
CAP	Community-Acquired Pneumonia
CI	Confidence Interval
COPD	Chronic Obstructive Pulmonary Disease
CRAB	Carbapenem-resistant *Acinetobacter baumannii*
CRP	C-Reactive Protein
eGFR	Estimated Glomerular Filtration Rate
EUCAST	European Committee on Antimicrobial Susceptibility Testing
HAP	Hospital-Acquired Pneumonia
ICU	Intensive Care Unit
IQR	Interquartile Range
KDIGO	Kidney Disease: Improving Global Outcomes
MDR	Multidrug-resistant
NLR	Neutrophil-to-Lymphocyte Ratio
OR	Odds Ratio
ROC	Receiver Operating Characteristic
SD	Standard Deviation
VAP	Ventilator-Associated Pneumonia
VIF	Variance Inflation Factor
WBC	White Blood Cell count
XDR	Extensively drug-resistant
